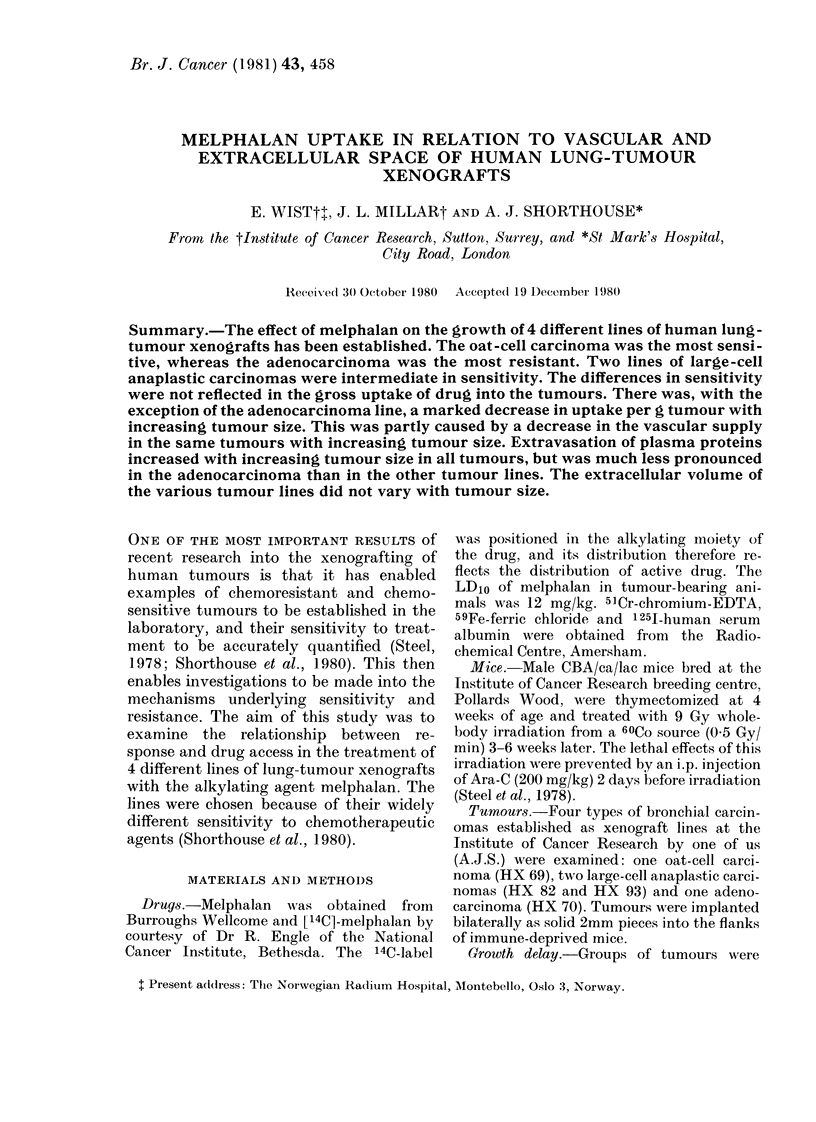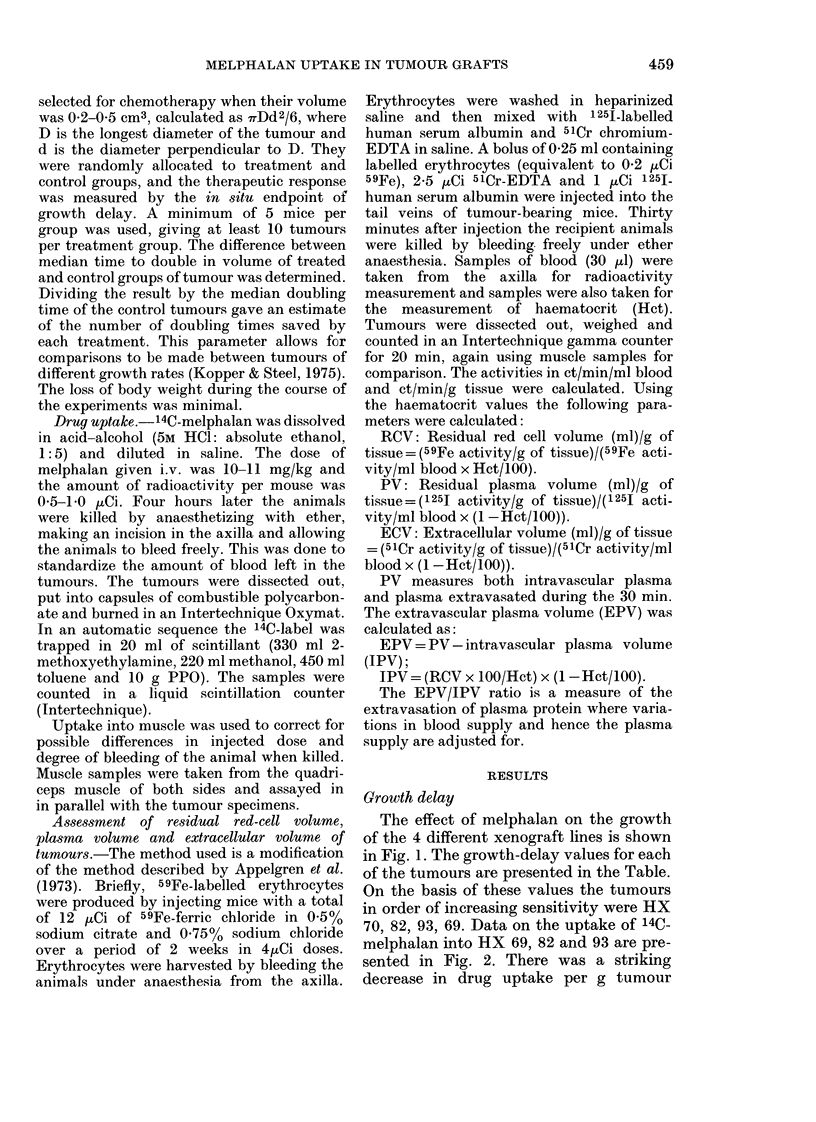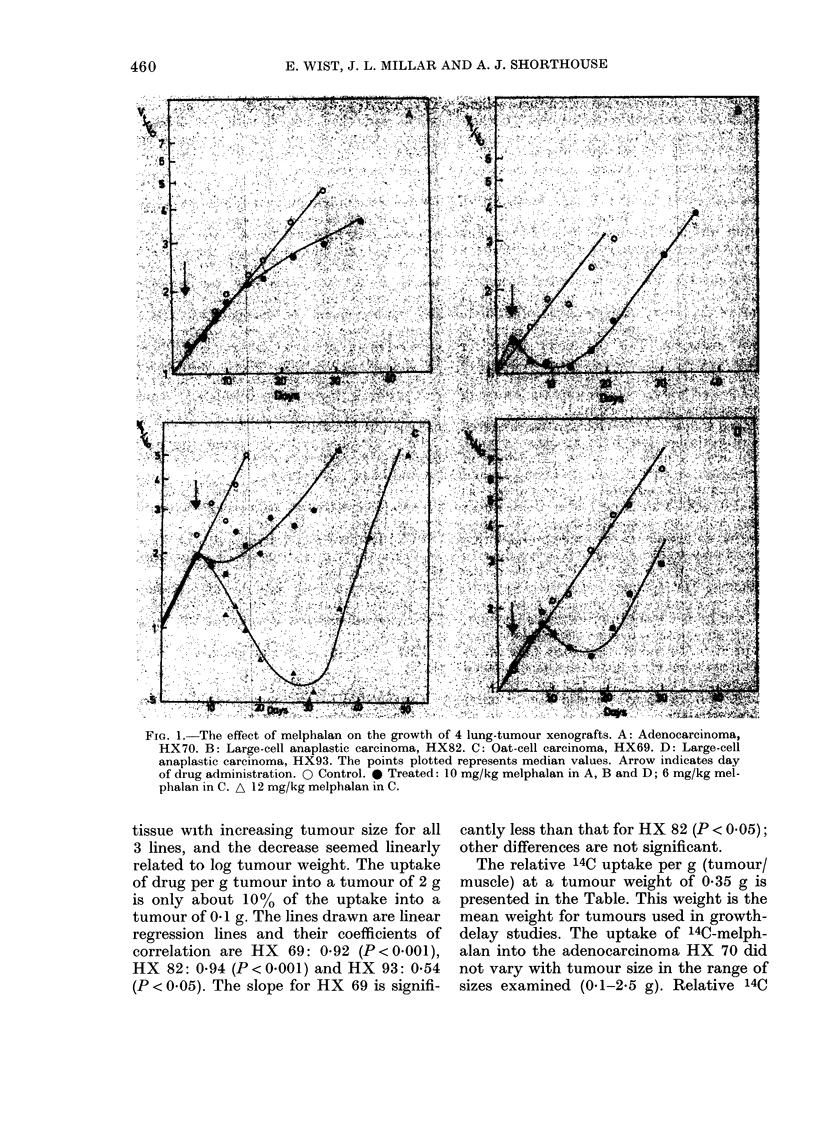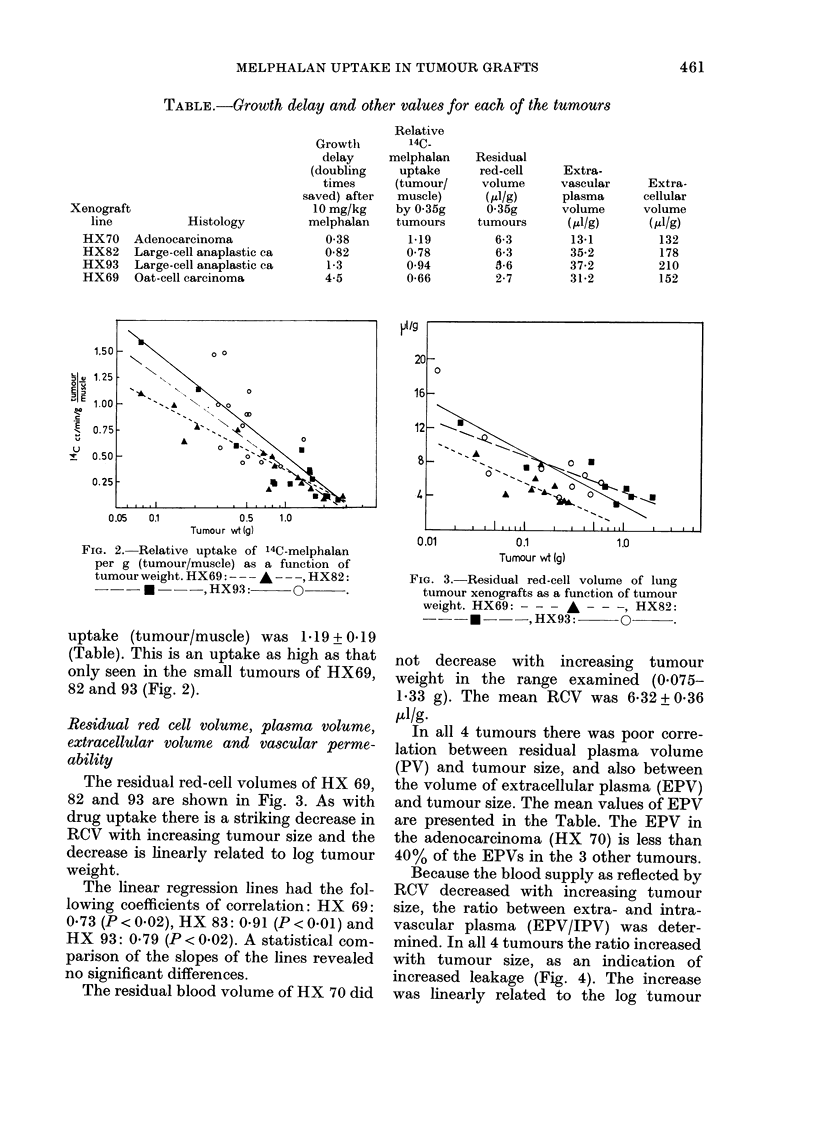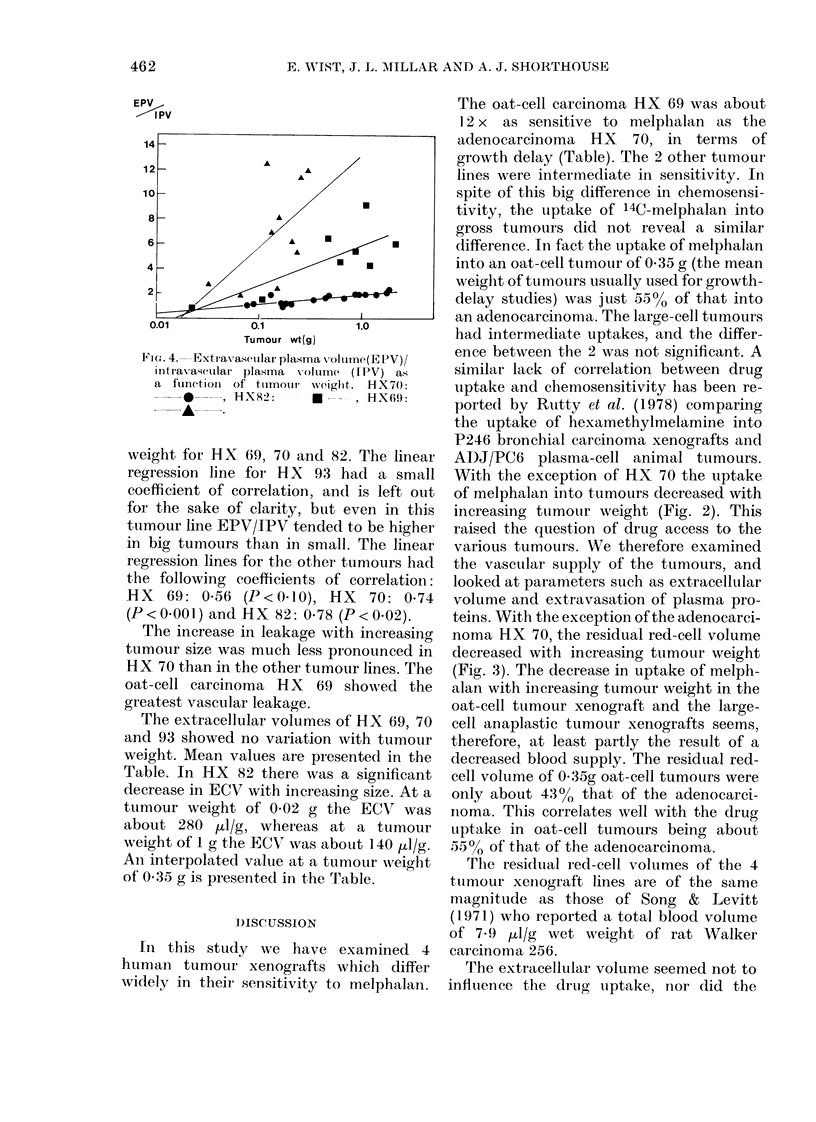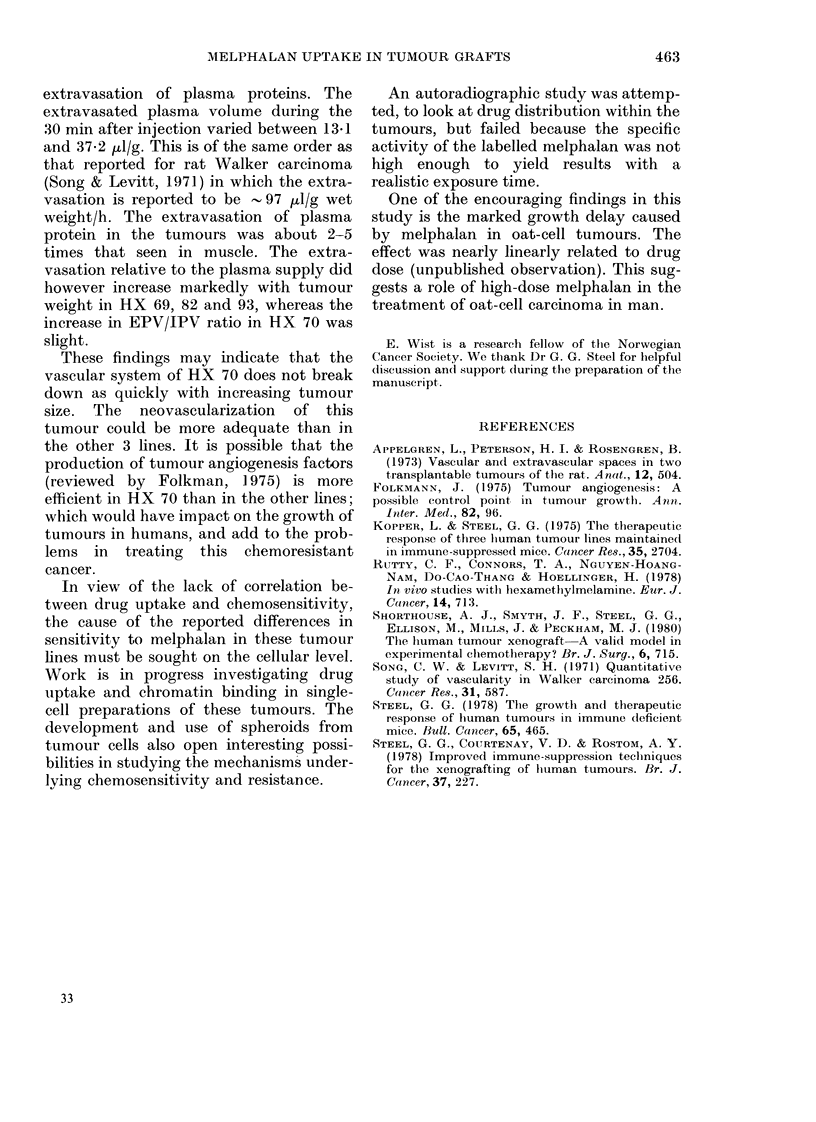# Melphalan uptake in relation to vascular and extracellular space of human lung-tumour xenografts.

**DOI:** 10.1038/bjc.1981.67

**Published:** 1981-04

**Authors:** E. Wist, J. L. Millar, A. J. Shorthouse

## Abstract

The effect of melphalan on the growth of 4 different lines of human lung-tumour xenografts has been established. The oat-cell carcinoma was the most sensitive, whereas the adenocarcinoma was the most resistant. Two lines of large-cell anaplastic carcinomas were intermediate in sensitivity. The differences in sensitivity were not reflected in the gross uptake of drug into the tumours. There was, with the exception of the adenocarcinoma line, a marked decrease in uptake per g tumour with increasing tumour size. This was partly caused by a decrease in the vascular supply in the same tumours with increasing tumour size. Extravasation of plasma proteins increased with increasing tumour size in all tumours, but was much less pronounced in the adenocarcinoma than in the other tumour lines. The extracellular volume of the various tumour lines did not vary with tumour size.


*Br. J. Cancer* (1981) **43**, 458

### MELPHALAN UPTAKE IN RELATION TO VASCULAR AND EXTRACELLULAR SPACE OF HUMAN LUNG-TUMOUR XENOGRAFTS

E. WIST†‡, J. L. MILLAR† AND A. J. SHORTHOUSE*

*From the †Institute of Cancer Research, Sutton, Surrey, and *St Mark's Hospital, City Road, London*

Received 30 October 1980 Accepted 19 December 1980

**Summary.—The effect of melphalan on the growth of 4 different lines of human lung-tumour xenografts has been established. The oat-cell carcinoma was the most sensitive, whereas the adenocarcinoma was the most resistant. Two lines of large-cell anaplastic carcinomas were intermediate in sensitivity. The differences in sensitivity were not reflected in the gross uptake of drug into the tumours. There was, with the exception of the adenocarcinoma line, a marked decrease in uptake per g tumour with increasing tumour size. This was partly caused by a decrease in the vascular supply in the same tumours with increasing tumour size. Extravasation of plasma proteins increased with increasing tumour size in all tumours, but was much less pronounced in the adenocarcinoma than in the other tumour lines. The extracellular volume of the various tumour lines did not vary with tumour size.**

One of the most important results of recent research into the xenografting of human tumours is that it has enabled examples of chemoresistant and chemosensitive tumours to be established in the laboratory, and their sensitivity to treatment to be accurately quantified (Steel, 1978; Shorthouse *et al.*, 1980). This then enables investigations to be made into the mechanisms underlying sensitivity and resistance. The aim of this study was to examine the relationship between response and drug access in the treatment of 4 different lines of lung-tumour xenografts with the alkylating agent melphalan. The lines were chosen because of their widely different sensitivity to chemotherapeutic agents (Shorthouse *et al.*, 1980).

### MATERIALS AND METHODS

*Drugs.*—Melphalan was obtained from Burroughs Wellcome and [$^{14}C$]-melphalan by courtesy of Dr R. Engle of the National Cancer Institute, Bethesda. The $^{14}C$-label was positioned in the alkylating moiety of the drug, and its distribution therefore reflects the distribution of active drug. The $LD_{10}$ of melphalan in tumour-bearing animals was 12 mg/kg. $^{51}Cr$-chromium-EDTA, $^{59}Fe$-ferric chloride and $^{125}I$-human serum albumin were obtained from the Radiochemical Centre, Amersham.

*Mice.*—Male CBA/ca/lac mice bred at the Institute of Cancer Research breeding centre, Pollards Wood, were thymectomized at 4 weeks of age and treated with 9 Gy whole-body irradiation from a $^{60}Co$ source (0·5 Gy/min) 3–6 weeks later. The lethal effects of this irradiation were prevented by an i.p. injection of Ara-C (200 mg/kg) 2 days before irradiation (Steel *et al.*, 1978).

*Tumours.*—Four types of bronchial carcinomas established as xenograft lines at the Institute of Cancer Research by one of us (A.J.S.) were examined: one oat-cell carcinoma (HX 69), two large-cell anaplastic carcinomas (HX 82 and HX 93) and one adenocarcinoma (HX 70). Tumours were implanted bilaterally as solid 2mm pieces into the flanks of immune-deprived mice.

*Growth delay.*—Groups of tumours were

‡ Present address: The Norwegian Radium Hospital, Montebello, Oslo 3, Norway.

selected for chemotherapy when their volume was 0·2–0·5 $cm^3$, calculated as $\pi Dd^2/6$, where D is the longest diameter of the tumour and d is the diameter perpendicular to D. They were randomly allocated to treatment and control groups, and the therapeutic response was measured by the *in situ* endpoint of growth delay. A minimum of 5 mice per group was used, giving at least 10 tumours per treatment group. The difference between median time to double in volume of treated and control groups of tumour was determined. Dividing the result by the median doubling time of the control tumours gave an estimate of the number of doubling times saved by each treatment. This parameter allows for comparisons to be made between tumours of different growth rates (Kopper & Steel, 1975). The loss of body weight during the course of the experiments was minimal.

*Drug uptake.*—$^{14}C$-melphalan was dissolved in acid–alcohol (5M HCl: absolute ethanol, 1:5) and diluted in saline. The dose of melphalan given i.v. was 10–11 mg/kg and the amount of radioactivity per mouse was 0·5–1·0 $\mu$Ci. Four hours later the animals were killed by anaesthetizing with ether, making an incision in the axilla and allowing the animals to bleed freely. This was done to standardize the amount of blood left in the tumours. The tumours were dissected out, put into capsules of combustible polycarbonate and burned in an Intertechnique Oxymat. In an automatic sequence the $^{14}C$-label was trapped in 20 ml of scintillant (330 ml 2-methoxyethylamine, 220 ml methanol, 450 ml toluene and 10 g PPO). The samples were counted in a liquid scintillation counter (Intertechnique).

Uptake into muscle was used to correct for possible differences in injected dose and degree of bleeding of the animal when killed. Muscle samples were taken from the quadriceps muscle of both sides and assayed in in parallel with the tumour specimens.

*Assessment of residual red-cell volume, plasma volume and extracellular volume of tumours.*—The method used is a modification of the method described by Appelgren *et al.* (1973). Briefly, $^{59}Fe$-labelled erythrocytes were produced by injecting mice with a total of 12 $\mu$Ci of $^{59}Fe$-ferric chloride in 0·5% sodium citrate and 0·75% sodium chloride over a period of 2 weeks in 4$\mu$Ci doses. Erythrocytes were harvested by bleeding the animals under anaesthesia from the axilla. Erythrocytes were washed in heparinized saline and then mixed with $^{125}I$-labelled human serum albumin and $^{51}Cr$ chromium-EDTA in saline. A bolus of 0·25 ml containing labelled erythrocytes (equivalent to 0·2 $\mu$Ci $^{59}Fe$), 2·5 $\mu$Ci $^{51}Cr$-EDTA and 1 $\mu$Ci $^{125}I$-human serum albumin were injected into the tail veins of tumour-bearing mice. Thirty minutes after injection the recipient animals were killed by bleeding freely under ether anaesthesia. Samples of blood (30 $\mu$l) were taken from the axilla for radioactivity measurement and samples were also taken for the measurement of haematocrit (Hct). Tumours were dissected out, weighed and counted in an Intertechnique gamma counter for 20 min, again using muscle samples for comparison. The activities in ct/min/ml blood and ct/min/g tissue were calculated. Using the haematocrit values the following parameters were calculated:

RCV: Residual red cell volume (ml)/g of tissue = ($^{59}Fe$ activity/g of tissue)/($^{59}Fe$ activity/ml blood × Hct/100).

PV: Residual plasma volume (ml)/g of tissue = ($^{125}I$ activity/g of tissue)/($^{125}I$ activity/ml blood × (1 − Hct/100)).

ECV: Extracellular volume (ml)/g of tissue = ($^{51}Cr$ activity/g of tissue)/($^{51}Cr$ activity/ml blood × (1 − Hct/100)).

PV measures both intravascular plasma and plasma extravasated during the 30 min. The extravascular plasma volume (EPV) was calculated as:

EPV = PV − intravascular plasma volume (IPV);

IPV = (RCV × 100/Hct) × (1 − Hct/100).

The EPV/IPV ratio is a measure of the extravasation of plasma protein where variations in blood supply and hence the plasma supply are adjusted for.

### RESULTS

### *Growth delay*

The effect of melphalan on the growth of the 4 different xenograft lines is shown in Fig. 1. The growth-delay values for each of the tumours are presented in the Table. On the basis of these values the tumours in order of increasing sensitivity were HX 70, 82, 93, 69. Data on the uptake of $^{14}C$-melphalan into HX 69, 82 and 93 are presented in Fig. 2. There was a striking decrease in drug uptake per g tumour

FIG. 1.—The effect of melphalan on the growth of 4 lung-tumour xenografts. A: Adenocarcinoma, HX70. B: Large-cell anaplastic carcinoma, HX82. C: Oat-cell carcinoma, HX69. D: Large-cell anaplastic carcinoma, HX93. The points plotted represents median values. Arrow indicates day of drug administration. ○ Control. ● Treated: 10 mg/kg melphalan in A, B and D; 6 mg/kg melphalan in C. △ 12 mg/kg melphalan in C.

tissue with increasing tumour size for all 3 lines, and the decrease seemed linearly related to log tumour weight. The uptake of drug per g tumour into a tumour of 2 g is only about 10% of the uptake into a tumour of 0·1 g. The lines drawn are linear regression lines and their coefficients of correlation are HX 69: 0·92 ($P < 0{\cdot}001$), HX 82: 0·94 ($P < 0{\cdot}001$) and HX 93: 0·54 ($P < 0{\cdot}05$). The slope for HX 69 is significantly less than that for HX 82 ($P < 0{\cdot}05$); other differences are not significant.

The relative $^{14}C$ uptake per g (tumour/muscle) at a tumour weight of 0·35 g is presented in the Table. This weight is the mean weight for tumours used in growth-delay studies. The uptake of $^{14}C$-melphalan into the adenocarcinoma HX 70 did not vary with tumour size in the range of sizes examined (0·1–2·5 g). Relative $^{14}C$

TABLE.—*Growth delay and other values for each of the tumours*

| Xenograft line | Histology | Growth delay (doubling times saved) after 10 mg/kg melphalan | Relative $^{14}C$-melphalan uptake (tumour/muscle) by 0·35g tumours | Residual red-cell volume (μl/g) 0·35g tumours | Extra-vascular plasma volume (μl/g) | Extra-cellular volume (μl/g) |
|---|---|---|---|---|---|---|
| HX70 | Adenocarcinoma | 0·38 | 1·19 | 6·3 | 13·1 | 132 |
| HX82 | Large-cell anaplastic ca | 0·82 | 0·78 | 6·3 | 35·2 | 178 |
| HX93 | Large-cell anaplastic ca | 1·3 | 0·94 | 5·6 | 37·2 | 210 |
| HX69 | Oat-cell carcinoma | 4·5 | 0·66 | 2·7 | 31·2 | 152 |

FIG. 2.—Relative uptake of $^{14}C$-melphalan per g (tumour/muscle) as a function of tumour weight. HX69: – – – ▲ – – –, HX82: ——— ■ ———, HX93: ——— ○ ———.

FIG. 3.—Residual red-cell volume of lung tumour xenografts as a function of tumour weight. HX69: – – – ▲ – – –, HX82: ——— ■ ———, HX93: ——— ○ ———.

uptake (tumour/muscle) was 1·19 ± 0·19 (Table). This is an uptake as high as that only seen in the small tumours of HX69, 82 and 93 (Fig. 2).

*Residual red cell volume, plasma volume, extracellular volume and vascular permeability*

The residual red-cell volumes of HX 69, 82 and 93 are shown in Fig. 3. As with drug uptake there is a striking decrease in RCV with increasing tumour size and the decrease is linearly related to log tumour weight.

The linear regression lines had the following coefficients of correlation: HX 69: 0·73 ($P < 0·02$), HX 83: 0·91 ($P < 0·01$) and HX 93: 0·79 ($P < 0·02$). A statistical comparison of the slopes of the lines revealed no significant differences.

The residual blood volume of HX 70 did not decrease with increasing tumour weight in the range examined (0·075–1·33 g). The mean RCV was 6·32 ± 0·36 μl/g.

In all 4 tumours there was poor correlation between residual plasma volume (PV) and tumour size, and also between the volume of extracellular plasma (EPV) and tumour size. The mean values of EPV are presented in the Table. The EPV in the adenocarcinoma (HX 70) is less than 40% of the EPVs in the 3 other tumours.

Because the blood supply as reflected by RCV decreased with increasing tumour size, the ratio between extra- and intravascular plasma (EPV/IPV) was determined. In all 4 tumours the ratio increased with tumour size, as an indication of increased leakage (Fig. 4). The increase was linearly related to the log tumour

FIG. 4.—Extravascular plasma volume(EPV)/ intravascular plasma volume (IPV) as a function of tumour weight. HX70: ——●——, HX82: ——■——, HX69: ——▲——.

weight for HX 69, 70 and 82. The linear regression line for HX 93 had a small coefficient of correlation, and is left out for the sake of clarity, but even in this tumour line EPV/IPV tended to be higher in big tumours than in small. The linear regression lines for the other tumours had the following coefficients of correlation: HX 69: 0·56 ($P<0·10$), HX 70: 0·74 ($P<0·001$) and HX 82: 0·78 ($P<0·02$).

The increase in leakage with increasing tumour size was much less pronounced in HX 70 than in the other tumour lines. The oat-cell carcinoma HX 69 showed the greatest vascular leakage.

The extracellular volumes of HX 69, 70 and 93 showed no variation with tumour weight. Mean values are presented in the Table. In HX 82 there was a significant decrease in ECV with increasing size. At a tumour weight of 0·02 g the ECV was about 280 $\mu$l/g, whereas at a tumour weight of 1 g the ECV was about 140 $\mu$l/g. An interpolated value at a tumour weight of 0·35 g is presented in the Table.

### DISCUSSION

In this study we have examined 4 human tumour xenografts which differ widely in their sensitivity to melphalan. The oat-cell carcinoma HX 69 was about 12× as sensitive to melphalan as the adenocarcinoma HX 70, in terms of growth delay (Table). The 2 other tumour lines were intermediate in sensitivity. In spite of this big difference in chemosensitivity, the uptake of $^{14}$C-melphalan into gross tumours did not reveal a similar difference. In fact the uptake of melphalan into an oat-cell tumour of 0·35 g (the mean weight of tumours usually used for growth-delay studies) was just 55% of that into an adenocarcinoma. The large-cell tumours had intermediate uptakes, and the difference between the 2 was not significant. A similar lack of correlation between drug uptake and chemosensitivity has been reported by Rutty *et al.* (1978) comparing the uptake of hexamethylmelamine into P246 bronchial carcinoma xenografts and ADJ/PC6 plasma-cell animal tumours. With the exception of HX 70 the uptake of melphalan into tumours decreased with increasing tumour weight (Fig. 2). This raised the question of drug access to the various tumours. We therefore examined the vascular supply of the tumours, and looked at parameters such as extracellular volume and extravasation of plasma proteins. With the exception of the adenocarcinoma HX 70, the residual red-cell volume decreased with increasing tumour weight (Fig. 3). The decrease in uptake of melphalan with increasing tumour weight in the oat-cell tumour xenograft and the large-cell anaplastic tumour xenografts seems, therefore, at least partly the result of a decreased blood supply. The residual red-cell volume of 0·35g oat-cell tumours were only about 43% that of the adenocarcinoma. This correlates well with the drug uptake in oat-cell tumours being about 55% of that of the adenocarcinoma.

The residual red-cell volumes of the 4 tumour xenograft lines are of the same magnitude as those of Song & Levitt (1971) who reported a total blood volume of 7·9 $\mu$l/g wet weight of rat Walker carcinoma 256.

The extracellular volume seemed not to influence the drug uptake, nor did the extravasation of plasma proteins. The extravasated plasma volume during the 30 min after injection varied between 13·1 and 37·2 $\mu$l/g. This is of the same order as that reported for rat Walker carcinoma (Song & Levitt, 1971) in which the extravasation is reported to be $\sim$97 $\mu$l/g wet weight/h. The extravasation of plasma protein in the tumours was about 2–5 times that seen in muscle. The extravasation relative to the plasma supply did however increase markedly with tumour weight in HX 69, 82 and 93, whereas the increase in EPV/IPV ratio in HX 70 was slight.

These findings may indicate that the vascular system of HX 70 does not break down as quickly with increasing tumour size. The neovascularization of this tumour could be more adequate than in the other 3 lines. It is possible that the production of tumour angiogenesis factors (reviewed by Folkman, 1975) is more efficient in HX 70 than in the other lines; which would have impact on the growth of tumours in humans, and add to the problems in treating this chemoresistant cancer.

In view of the lack of correlation between drug uptake and chemosensitivity, the cause of the reported differences in sensitivity to melphalan in these tumour lines must be sought on the cellular level. Work is in progress investigating drug uptake and chromatin binding in single-cell preparations of these tumours. The development and use of spheroids from tumour cells also open interesting possibilities in studying the mechanisms underlying chemosensitivity and resistance.

An autoradiographic study was attempted, to look at drug distribution within the tumours, but failed because the specific activity of the labelled melphalan was not high enough to yield results with a realistic exposure time.

One of the encouraging findings in this study is the marked growth delay caused by melphalan in oat-cell tumours. The effect was nearly linearly related to drug dose (unpublished observation). This suggests a role of high-dose melphalan in the treatment of oat-cell carcinoma in man.

E. Wist is a research fellow of the Norwegian Cancer Society. We thank Dr G. G. Steel for helpful discussion and support during the preparation of the manuscript.

### REFERENCES

Appelgren, L., Peterson, H. I. & Rosengren, B. (1973) Vascular and extravascular spaces in two transplantable tumours of the rat. *Anat.*, **12**, 504.

Folkmann, J. (1975) Tumour angiogenesis: A possible control point in tumour growth. *Ann. Inter. Med.*, **82**, 96.

Kopper, L. & Steel, G. G. (1975) The therapeutic response of three human tumour lines maintained in immune-suppressed mice. *Cancer Res.*, **35**, 2704.

Rutty, C. F., Connors, T. A., Nguyen-Hoang-Nam, Do-Cao-Thang & Hoellinger, H. (1978) *In vivo* studies with hexamethylmelamine. *Eur. J. Cancer*, **14**, 713.

Shorthouse, A. J., Smyth, J. F., Steel, G. G., Ellison, M., Mills, J. & Peckham, M. J. (1980) The human tumour xenograft—A valid model in experimental chemotherapy? *Br. J. Surg.*, **6**, 715.

Song, C. W. & Levitt, S. H. (1971) Quantitative study of vascularity in Walker carcinoma 256. *Cancer Res.*, **31**, 587.

Steel, G. G. (1978) The growth and therapeutic response of human tumours in immune deficient mice. *Bull. Cancer*, **65**, 465.

Steel, G. G., Courtenay, V. D. & Rostom, A. Y. (1978) Improved immune-suppression techniques for the xenografting of human tumours. *Br. J. Cancer*, **37**, 227.